# Soil microbial inoculation during flood events shapes headwater stream microbial communities and diversity

**DOI:** 10.1007/s00248-021-01700-3

**Published:** 2021-02-02

**Authors:** Florian Caillon, Katharina Besemer, Peter Peduzzi, Jakob Schelker

**Affiliations:** 1 WasserCluster Lunz/Biological Station GmbH, A-3293, Lunz am See, Austria; 2 Division of Limnology, Department of Functional and Evolutionary Ecology, University of Vienna, A-1090 Vienna, Austria

**Keywords:** Soil inoculation, Soil bacteria, Microbial diversity, Sequencing

## Abstract

Flood events are now recognized as potentially important occasions for the transfer of soil microbes to stream ecosystems. Yet, little is known about these “dynamic pulses of microbial life” for stream bacterial community composition (BCC) and diversity. In this study, we explored the potential alteration of stream BCC by soil inoculation during high flow events in six pre-alpine first order streams and the larger Oberer Seebach. During 1 year, we compared variations of BCC in soil water, stream water and in benthic biofilms at different flow conditions (low to intermediate flows versus high flow). Bacterial diversity was lowest in biofilms, followed by soils and highest in headwater streams and the Oberer Seebach. In headwater streams, bacterial diversity was significantly higher during high flow, as compared to low flow (Shannon diversity: 7.6 versus 7.9 at low versus high flow, respectively, *p* < 0.001). Approximately 70% of the bacterial operational taxonomic units (OTUs) from streams and stream biofilms were the same as in soil water, while in the latter one third of the OTUs were specific to high flow conditions. These soil high-flow OTUs were also found in streams and biofilms at other times of the year. These results demonstrate the relevance of floods in generating short and reoccurring inoculation events for flowing waters. Moreover, they show that soil microbial inoculation during high flow enhances microbial diversity and shapes fluvial BCC even during low flow. Hence, soil microbial inoculation during floods could act as a previously overlooked driver of microbial diversity in headwater streams.

## Introduction

Streams and rivers maintain large microbial communities that are essential for the functioning of fluvial ecosystems. Microbial communities in streams contribute significantly to biogeochemical cycles of essential nutrients, such as carbon and nitrogen that they respire, convert and metabolize [1], drive organic matter decomposition [2], and carbon dioxide evasion [3], and are critical for the transfer of carbon to higher trophic levels. Moreover, the capacity of aquatic microbes to reduce nutrient contamination often defines the suitability of water resources for the human use [4].

Stream microbial diversity is enormous [5] and varies depending on the position in the aquatic continuum [6, 7]. In headwater streams, the furthest upstream tributaries in river networks, microbial community diversity is high and has been partly found to resemble soil microbial communities [8, 9]. This is likely caused by significant transport of soil bacteria from catchment soils into headwater streams [10–12]. Further downstream, bacterial richness has been observed to decrease while the proportion of typical freshwater taxa increases [13, 14]. As directional dispersal occurs within the dendritic structure of river networks [15, 16], headwater streams have been named as reservoirs of microbial diversity of river network metacommunities [5].

A large part of the fluvial freshwater microbiome lives attached to surface biofilms [17, 18]. Biofilms are stable physical structures in which bacteria live together with algae and other microbes [17]. Although stream biofilms are assumed to assemble from the suspended bacterial community present in the water column, biofilm microbial assemblages are noticeably different from free-flowing communities [19]. As processes such as species sorting reduce stream biofilm diversity through competition, the biofilm diversity is believed to be maintained by a continuous inflow of microorganisms from upstream catchments [19, 20]. Vice versa, stream biofilms may also promote the dispersal of aquatic bacterial species within aquatic networks, for example when biofilms release microbes and thereby reinoculate the free-flowing stream community with these taxa [17].

Bacterial communities in lotic systems show variations in their taxonomic composition over time in response to habitat conditions [21]. Environmental variables such as nutrient availability [22], water temperature [23], organic matter availability [24], and, notably, water flow [25] can all influence community composition. However, the temporal dynamics of water flows affect not only the biophysicochemical conditions within fluvial systems, but also the direct transfer of microbes from the catchment soils into streams [10].

Soils are connected to river networks by water flows that enter streams through the riparian zone [26, 27]. This connection is highly dynamic and is controlled by soil moisture [28] and precipitation [27]. Through this connection, soils provide nutrients, organic carbon, and microbes to the stream ecosystem [2, 11, 13, 25]. Soil bacterial abundance and diversity is large, with some estimates suggesting up to 10^6^ bacterial species in one gram of soil [29]. Thus, soils provide a potentially large source of microbial diversity for stream ecosystems, if soils become transiently connected to streams during hydrological events.

Previous work questioned if soil bacteria would be capable to cross the terrestrial-freshwater interface and successfully establish populations within freshwater ecosystems [30]. However, other findings suggest that at least some microbes originating from soils can thrive in freshwater environments [31]. For example, Fenchel et al. [32] found that soil microbes could develop in interstitial waters between soils particles. Further, more recent studies recognized a high overlap between the microbial communities of soils and freshwater environments [8, 9, 33]. Yet despite these notions, there is to date little knowledge on the precise spatiotemporal organization of soil microbial inoculation of stream ecosystems.

In this study, we aimed to explore the effects of the changes in the connectivity of catchment soils and streams caused by hydrological dynamics for the assembly of stream bacterial communities. Given the previously observed elevated bacterial abundances in streams during high flow events [10], we propose that soil microbial inoculation of streams takes place primarily during high flow events, when soil water contributions to streams are highest. We hypothesized that bacterial diversity in streams will be higher during high flow conditions, as compared to low flow. Furthermore, we proposed that bacterial communities in soils and streams homogenize at high flow due to an increase in soil contributions to small streams during floods. We evaluated these hypotheses by comparing bacterial community composition and diversity in streams and soil water under different flow conditions.

## Methods

### Study site and sampling

**Fig. 1:**
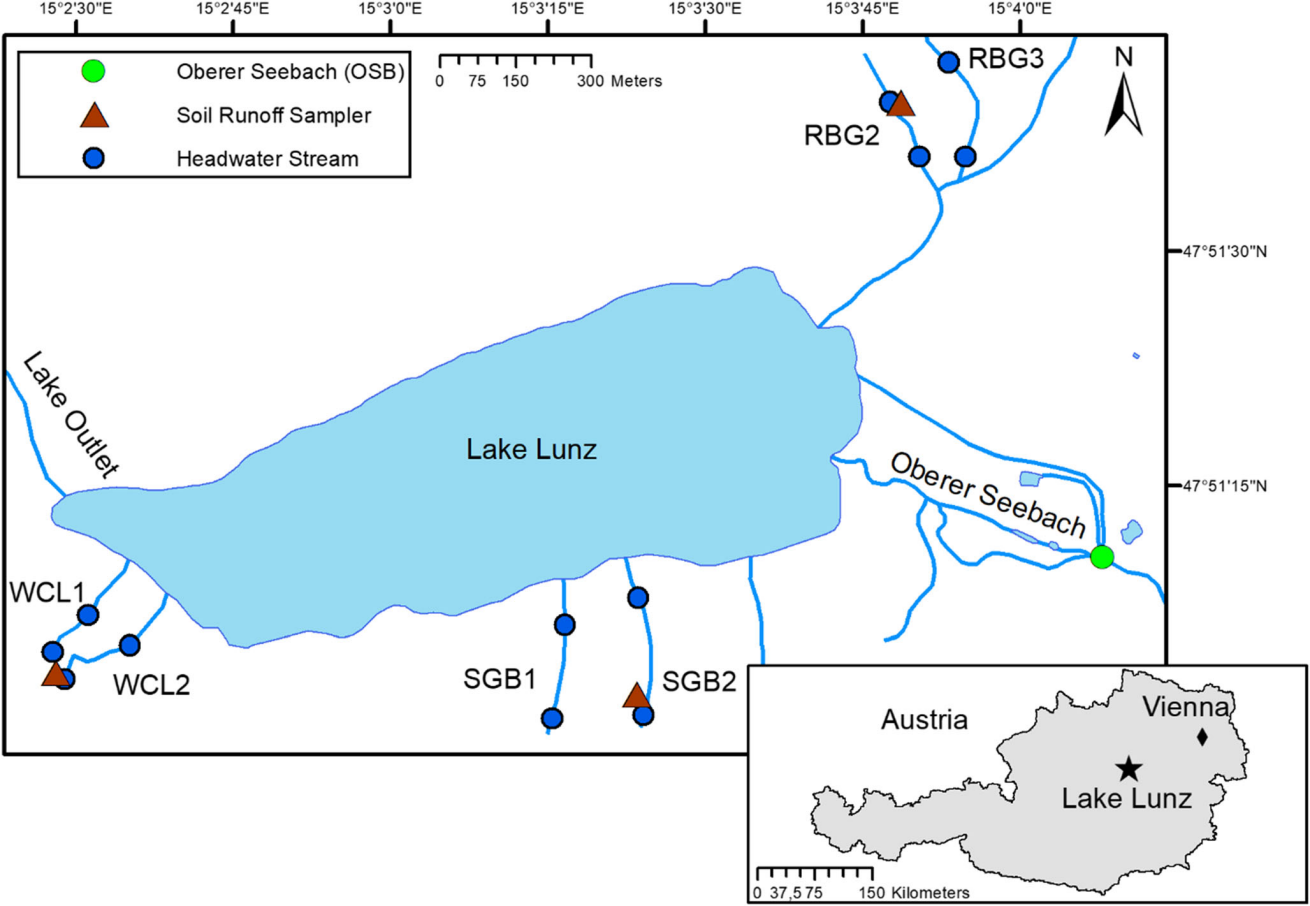
Locations of sampled soil water, headwater streams and Oberer Seebach near Lake Lunz in Austria. Dots represent stream sampling stations and brown triangles soil runoff samplers

We sampled soil water, stream water, and benthic biofilms once a month and during high flow events over a period of five months, resulting in six sampling occasions, including two high flow events. Sampling was carried out in the Oberer Seebach Catchment, around Lake Lunz in the eastern Alps near Lunz am See, Austria (Fig. 1). This catchment is largely pristine and the Oberer Seebach (OSB) is a well-studied stream with meteorological and discharge data available [34, 35]. The catchment is dominated by mixed deciduous forest with some meadows used for low intensity cattle grazing during summer. For this study, we selected three different hillslopes of the catchment (Rehberg, RBG; Schlögelberg, SBG; and the WasserCluster Lunz slope, WCL), each located within a radius of 1.5 km around Lake Lunz to ensure equal weather conditions (i.e., air temperature, precipitation). On each hillslope, we sampled two headwater streams (Strahler order 1) and soil water from two different depths (around 20 cm and around 50 cm deep). All headwater streams have similar width (< 1 m), depth (< 10 cm) and slopes (mean 29%) and are reasonably fast flowing (flow velocity 0.1 m s^−1^ at intermediate flow conditions; [10]).

We drew soil water samples from soil runoff samplers, which were installed in wet locations in close vicinity (< 15 m) to the source of one headwater stream on each hillslope. Each soil sampler consisted of two stainless steel soil water collectors of 2 m width that were pressed laterally into the hillslope (30–40 cm) at the respective depth [10]. Soil depth was not considered in this study, and both samples were used as replicates. The samplers intercept soil water from the unsaturated zone of the soil profile as it drains downwards along the hillslope, essentially representing mobile soil water on its way towards the stream. We performed the sampling by placing acid washed and precombusted (4 h at ~ 450 °C) 500-mL Schott bottles below the outflow of each sampler the day before sampling. During the driest summer conditions, some soil runoff samplers remained dry, reducing the number of soil water samples to 18.

In all six headwater streams, we collected stream samples at two different locations, one near the source of the stream (and the soil runoff samplers) and one ~ 400–500 m further downstream. Water samples were collected using pre-combusted (4 h at ~ 450 °C) 500-mL Schott Bottles and sterile 15-mL syringes when necessary.

Additionally, we collected water from a larger stream, the OSB (Strahler order 3) at three different locations across the width of the stream. This stream was sampled in order to compare its bacterial community to the communities upstream in the headwaters and because of the extensive data available for it.

Finally, we also sampled biofilms from the OSB due to their implication for bacteria dispersal and the potential impact of soil inoculation on their communities. Sterile ceramic tiles (5 by 5 cm), glued on bricks to prevent them from erosion, served as substratum for biofilm growth in the stream. Tiles were installed on the streambed 4 weeks prior to sampling to allow for biofilm growth. During flood events, the water level of the OSB was too high to allow for the collection of the tiles and, therefore, we could only sample one biofilm sample at high flow conditions. After sampling, tiles were kept at − 20 °C in sterile plastic bags until further processing.

In total, we collected 96 samples for the characterization of bacterial communities at different flow conditions (soil water *n* = 18, headwater streams *n* = 58, OSB water *n* = 14, and OSB biofilms *n* = 6).

### DNA extraction, PCR and Illumina sequencing

Water samples were kept at 4 °C and, within 10 h, were filtered in the lab on 0.2-μm mixed cellulose filters (Whatman ME24) and stored at − 20 °C in 15-mL sterile tubes until further processing. We extracted DNA from the filters and the biofilms using the DNeasy PowerSoil extraction kit (Qiagen) following the manufacturer’s protocol. This kit has been used successfully in earlier studies on stream water and biofilms [e.g., 24]. Prior to our extraction, we cut the filters into pieces using ethanol-flamed tweezers and scissors, biofilms were removed from the tiles using ethanol-flamed razor blades and spatula. The V3–V4 region of the 16S rRNA gene was amplified using the bacteria specific primers 341F (5’-CCT ACG GGN GGC WGC AG-3’) and 785R (5’-GAC TAC HVG GGT ATC TAA KCC-3’) [36]. PCR amplification, Illumina MiSeq library preparation and sequencing using V3 chemistry and 2 × 300 bp paired-end reads was carried out by LGC Genomics GmbH (Berlin, Germany). Sequences are accessible at the National Center for Biotechnology Information Sequence Read Archive under the accession number PRJNA685744.

### Bioinformatics

We processed amplicon sequences as in Cholet et al. [37]. The first steps followed the recommendations of Schirmer et al. [38]. We used sickle v1.2 to perform quality trimming of paired-end reads using a 20-bp sliding window. We trimmed reads where the average quality score dropped below 20 and discarded reads below 10-bp length. Then, we applied BayesHammer [39] from the Spades v2.5.0 assembler [40] to error correct the paired-end reads. Next, we used PANDAseq v2.4 [41] to assemble overlapping forward and reverse reads using a minimum overlap of 10 bp.

We followed the VSEARCH v2.3.4 pipeline for construction of Operational Taxonomic Unit (OTU) [42]. The pooled sequences were dereplicated, sorted and singletons discarded. We clustered sequences on a 97% identity level. We also performed de novo chimera detection by searching for potential parent reads, followed by reference-based chimera detection using the Silva aligned version of the gold database (https://www.mothur.org/w/images/f/f1/Silva.gold.bacteria.zip). Then we matched the original barcoded sequences against the clean OTUs with a 97% similarity threshold to generate OTU tables. We derived the taxonomic assignment by classification against the SILVA SSU Ref NR v123 database at a 90% confidence threshold. OTUs that occurred less than 10 times in the dataset, were classified as chloroplasts, or were not classified as bacteria, were excluded from further analysis. Also, one biofilm sample had a low number of reads (85) and was removed from the dataset. The cleaned dataset consisted of 7,418,358 sequences clustered into 25,985 OTUs. All OTUs could be classified to a phylum or lineage.

### Hydrology

Variability in stream runoff was analyzed using data from the OSB station at the inlet of Lake Lunz [34, 35]. Stream discharge (Q in m^3^ s^−1^) was derived from 10-min water level measurements by using well established rating curves [35]. From this data, daily average discharge was calculated and used for further analysis. As some data was missing due to malfunctioning of logger systems, missing daily discharge values were estimated from the gage at the lake outlet (*R*^2^ = 0.91 for daily Q’s from both stations).

Flow conditions were classified by the level of stream discharge similar to earlier work. In short, daily average discharge higher than 90% of the time in our study period (*Q* > 1.86 m^3^ s^−1^) was defined as high flow [10, 35]. Low flow was defined as all discharges lower than high flow (*Q* ≤ 1.86 m^3^ s^−1^) and thus effectively also includes intermediate flow conditions [10].

### Statistical analysis

Statistical analyses were performed in R with the *phyloseq*, *stats*, and *vegan* packages [43–45]. Nonmetric multidimensional scaling with Bray-Curtis distances was used to ordinate the samples based on their dissimilarity in community composition. ANOSIM analysis was carried out with 9999 permutations to test the differences between habitat type and flow conditions.

Richness and Shannon diversity (the number equivalent to the Shannon entropy; [46]) were calculated after rarefying to the lowest number of reads obtained from a sample (9905 reads) to account for differences in sequencing effort. Shapiro-Wilk test for normal distribution on the data indicated that richness was normally distributed while Shannon diversity was not. ANOVA and Tukey tests were used to compare the difference in richness between sample types and flow conditions. Mann–Whitney *U* tests and Kruskal–Wallis tests were used for Shannon diversity.

To identify potential habitat specialists and the impact of flow conditions on their dispersal, we calculated indicator values for each OTU using the *labdsv* R package [47]. We considered indicator species (in our case OTUs) as significant for indicator values > 0.7 and *p* values < 0.05. Additionally, we carried out a fast expectation-maximization for microbial source tracking (FEAST) analysis on bacteria using the *FEAST* R package. This analysis allows estimating the contribution of one habitat as a source, to another as a sink [48].

## Results

### Similarities in microbial communities across habitats

**Fig. 2:**
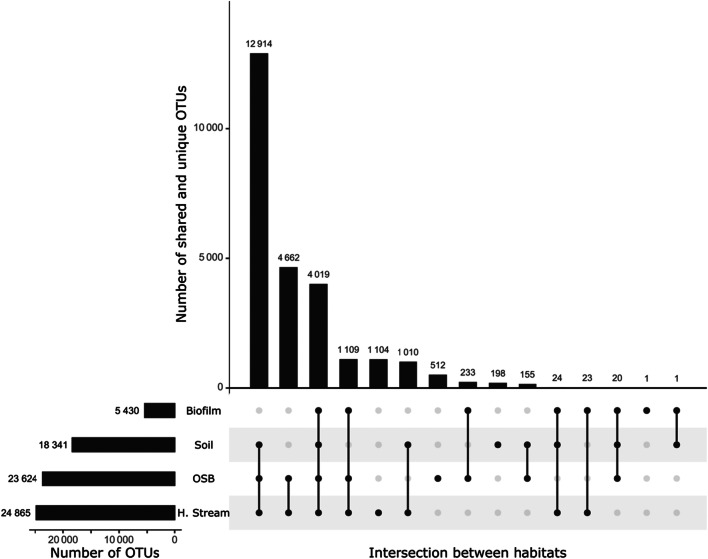
Number of shared and unique OTUs between soil water, headwater streams (h. streams), OSB water and biofilms bacterial communities. The total number of OTUs detected in each habitat is represented in the horizontal histogram. The vertical histogram represents the size of the intersection (the number of shared OTUs) between the habitats that are connected in the lower panel

In total, we recovered 25,985 OTUs of which 18,341 were present in soil water, 24,865 in stream water, 23,624 in the OSB water and 5430 in the biofilms. When we compared the different habitats, a large overlap was observed in the bacterial OTUs present in streams and the catchment soils (Fig. 2). Headwater streams, the OSB, and soil water shared 12,914 common OTUs. Further, we found that 72% of OTUs found in headwater streams, 72% of OTUs found in the OSB, and 75% of OTUs found in biofilms were also present in soil water. A strong overlap between bacterial communities was also observed between headwater streams and the OSB. These two habitats had 22,704 OTUs in common, which represents 91.3% of the headwater stream OTUs and 96.1% of the OSB OTUs. Biofilm communities represented a subset of the OSB OTUs, with only 49 OTUs occurring in biofilms but not in the OSB water.

### Bacterial diversity

Bacterial richness varied strongly across the landscape (ANOVA, *p* < 0.001). In soil water samples, after rarefying the data, between 1,396 and 9,685 OTUs were identified (mean (± SD) 5,112 ± 2,369). In headwater streams, 2,589 to 14,497 OTUs were found (mean 8,032 ± 2,553), while in the OSB 2,591 to 16,606 OTUs (mean 9,428 ± 4,173) were present. In stream biofilms between 728 and 3,133 (mean of 2,098 ± 959), OTUs were identified and this habitat presented a substantially lower richness than all other habitats.

Flow conditions had a significant effect on richness. During high flow events, the overall richness increased significantly by 37% (ANOVA, *p* < 0.001), from 6352 ± 2764 to 8716 ± 3455 OTUs as compared to low flow conditions. The strongest increase was measured in the OSB where the richness increased by 92% (ANOVA, *p* < 0.001) during high flow as compared to low flow. In contrast, we observed no difference in richness in soils and in biofilm samples during high flow versus low flow conditions, respectively.

**Fig. 3:**
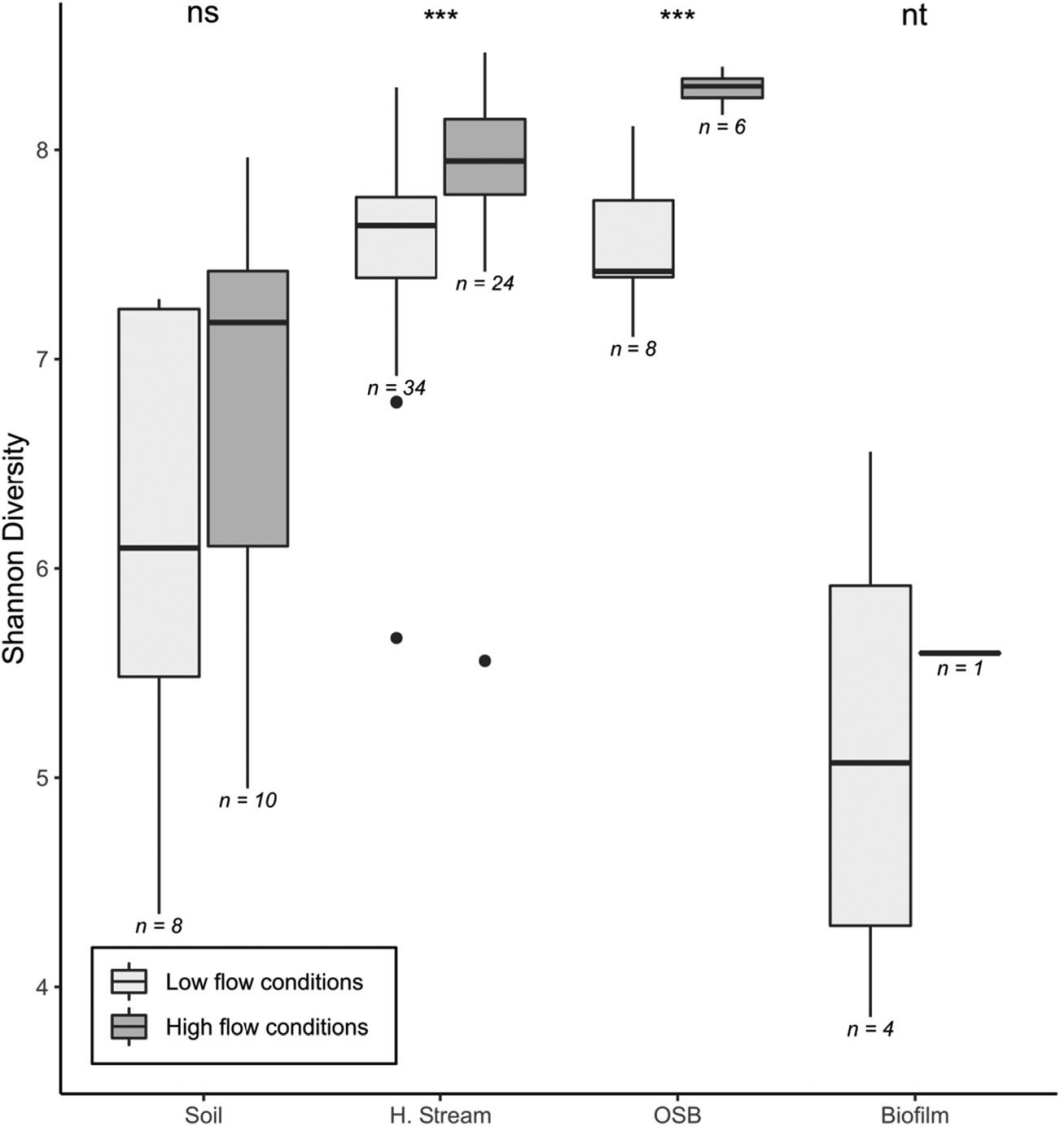
Shannon diversity index for each habitat of the soil-stream-river interface. Shannon diversity was calculated for high and low flow conditions. Horizontal lines show the median, boxes the 25th to 75th percentiles, whiskers the 5th and 95th percentile range. Black points are values outside the interquartile range. Values presented bellow each boxplot refer to sample size. Test results presented at the top refer to the high versus low flow comparisons of each compartment; ‘ns’ denotes not significant, ‘nt’ not tested, and ‘***’ a highly significant difference (Mann-Whitney *U*, *p* < 0.001). No statistical test was conducted on biofilm samples since there was only one biofilm sample at high flow. H. stream stands for headwater streams

We found Shannon diversity to be overall significantly higher in headwater streams and the OSB compared to soils and biofilm samples (Kruskal-Wallis, *p* < 0.001) (Fig. 3). During storms, the overall Shannon diversity increased significantly by 5% (Kruskal-Wallis, *p* < 0.001), from 7.2 ± 1.0 to 7.6 ± 1.0 as compared to low flow conditions. Shannon diversity did not appear to change with flow conditions in soils (Mann–Whitney *U*, *p* = 0.32) and biofilms individually. However, we quantified a significant increase in diversity in headwater streams and the OSB at high flow (+ 4% and + 10% respectively, Mann–Whitney *U*, *p* < 0.001).

**Fig. 4:**
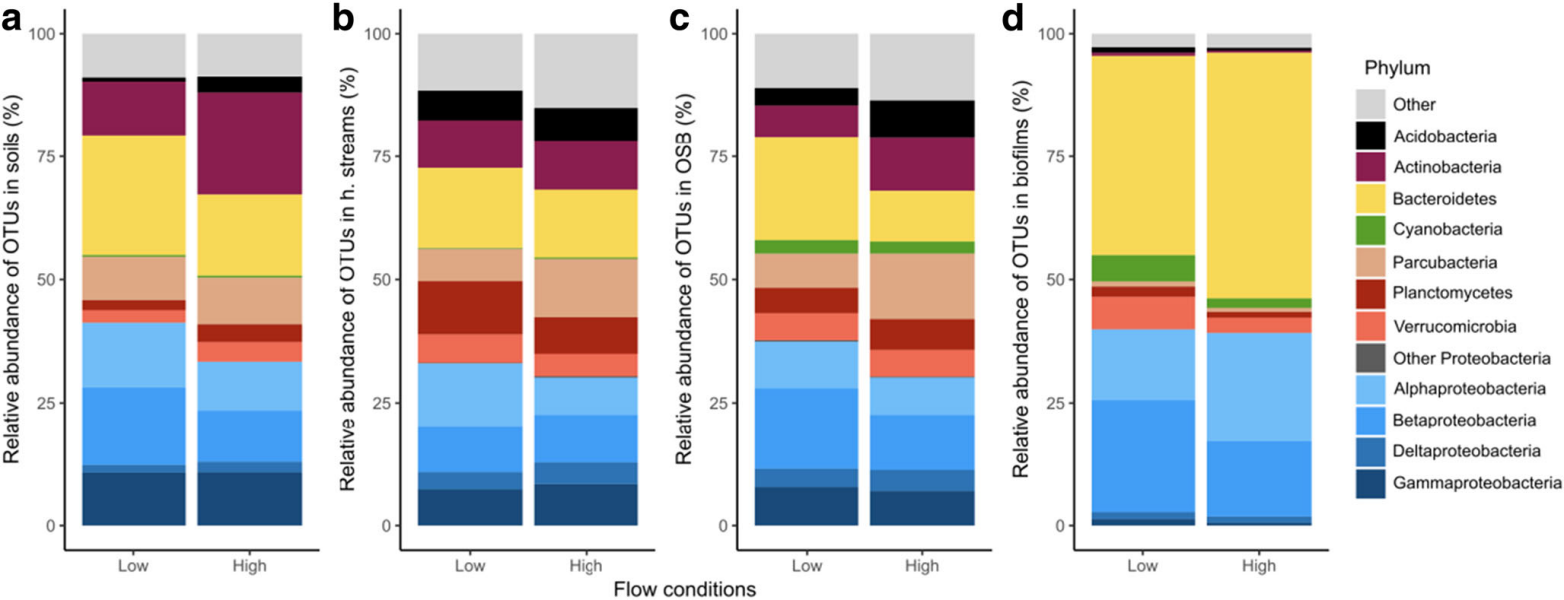
Bacterial community composition expressed as fraction of total bacterial sequences in soil water (**a**), headwater streams (**b**), the OSB (**c**) and biofilm samples (**d**), during high and low flow, respectively. Displayed are the 12 most abundant phyla, the remaining phyla were classified as ‘Other’

Bacterial communities varied in composition across soil water, streams and biofilm samples. While biofilm was dominated primarily by Bacteroidetes (42%, Fig. 4), soil and stream water was dominated by Bacteroidetes, Alpha- and Betaproteobacteria, Actinobacteria and a diverse set of less abundant phyla. Bacterial communities of headwater streams and OSB showed a higher proportion of Acidobacteria compared to soil water (Fig. 4). Bacteroidetes were the most abundant taxa in all habitats (20% in soil water, 15% in headwater streams, 16% in OSB, and 42% of biofilm sequences). Betaproteobacteria were more abundant in soil water than in headwater stream water and were more important under low than under high flow conditions. Alpha- and Gammaproteobacteria were also more important in soil water than in the stream water but showed no pattern with flow conditions. Parcubacteria presented similar proportions in soils, headwater streams and OSB (around 9% of bacterial sequences) and were almost absent from biofilm communities (1% of sequences). Their proportion increased in all habitats except biofilms during high flow conditions.

The indicator species analysis identified 196, 110, 124, and 189 significant indicator OTUs (*p* < 0.05) for soils, headwater streams, the OSB, and biofilms, respectively. Soil indicator OTUs were predominantly Bacteroidetes, Actinobacteria, and Betaproteobacteria (32%, 25%, and 21%, respectively). Headwater stream indicator OTUs were mostly identified as Planctomycetes and Bacteroidetes (28% and 25%, respectively). Indicator OTUs of the OSB were Cyanobacteria and Betaproteobacteria (29% and 22%, respectively). Finally, biofilm indicator OTUs were predominantly Bacteroidetes and Alphaproteobacteria (56% and 17%, respectively).

**Fig. 5:**
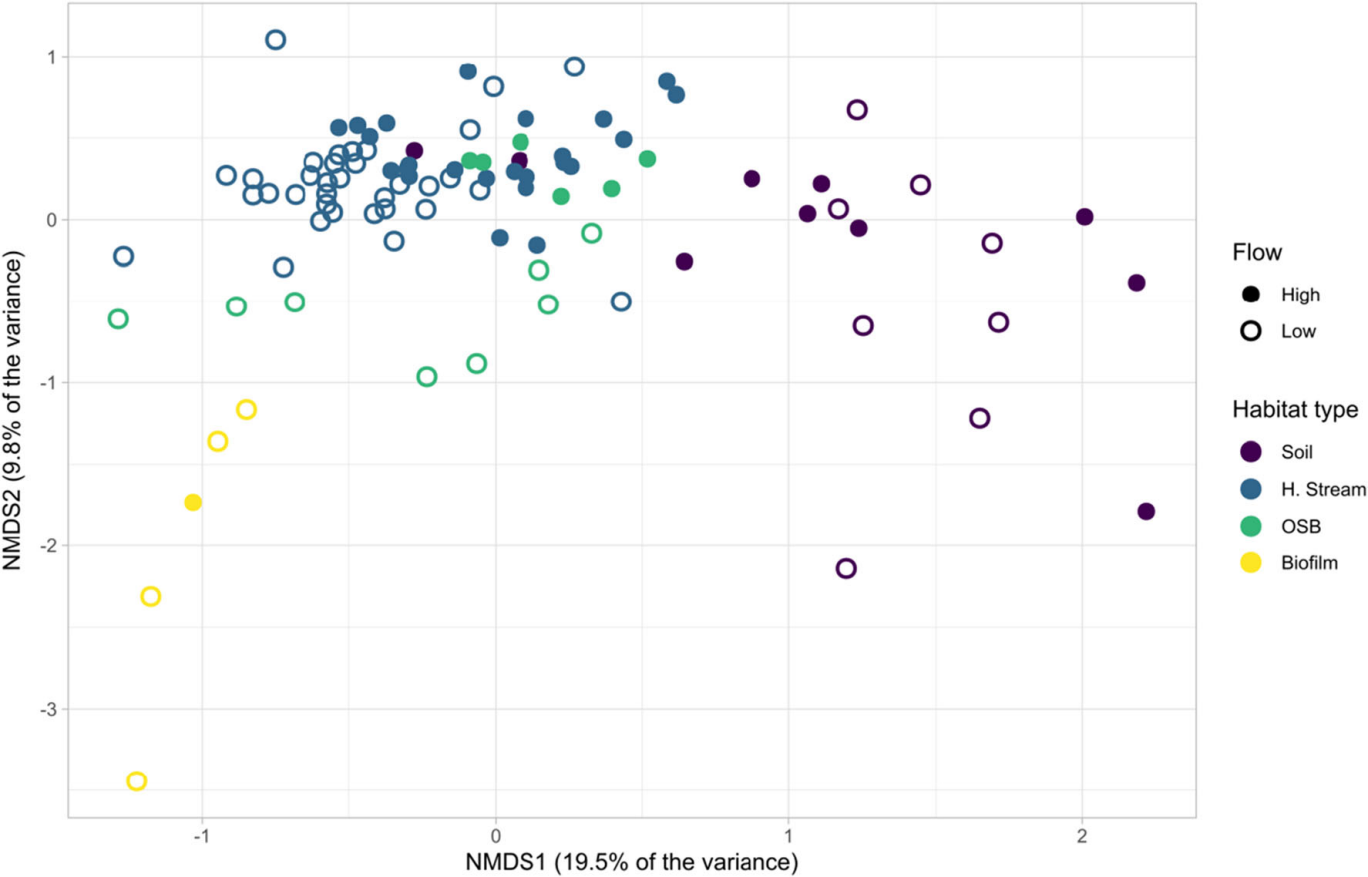
Nonmetric multidimensional scaling of bacterial communities of different habitat types and high and low flow conditions. H. stream stands for headwater streams

During high flow events, community composition changed in all habitats (Fig. 4). Nonmetric multidimensional scaling analysis showed that there was a statistical difference between bacterial communities based on habitat type (Fig. 5, ANOSIM, *R* = 0.71, *p* < 0.001). Overall, we found no difference in bacterial communities between high flow and low flow (ANOSIM, *R* = 0.09, *p* < 0.001) but within groups, small stream samples and the OSB showed a significant difference in their bacterial communities between high flow and low flow conditions (ANOSIM, *R* = 0.29 and 0.41, *p* < 0.001, and *p* = 0.005, respectively). At low flow, we observed a higher dissimilarity between habitat types in terms of bacterial communities than at high flow (ANOSIM, *R* = 0.87 and 0.50, respectively, *p* < 0.001), during which communities were more homogeneous between habitats.

### Contribution of soil taxa to downstream aquatic habitats

At low flow, 189 out of 196 soil indicative OTUs were also found in headwater streams and 178 in the OSB. At high flow, this number increased to 196 soil indicative OTUs in headwater streams and 189 in the OSB.

Similarly, the FEAST analysis confirmed the contribution of soil water to the downstream habitats. Soils as sources contributed 77% to headwater streams and OSB communities, and 83% to biofilm communities. When the other habitats were considered as additional sources besides soil water, the contribution of headwater streams to OSB communities and of OSB to biofilm communities were substantially lower (28% and 21%, respectively).

**Fig. 6:**
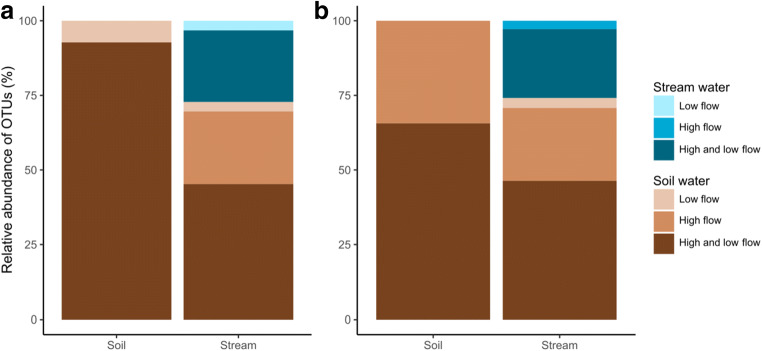
Relative abundance of OTUs in soil water and headwater streams at high flow (**a**) and low flow (**b**) categorized by the farthest upslope habitat and flow conditions they were first detected in. Values are expressed as a fraction of the total OTUs for each habitat

To further investigate the relevance of soil inoculation for streams and biofilm, each OTU was assigned to the most upstream environment and the flow conditions it was detected in. With this analysis (Fig. 6), we found that 72% of the bacterial OTUs present in small streams and the OSB and 75% of OTUs present in stream biofilms were first detected in soil water. The approximate proportion of these soil OTUs in downstream environments did not change with flow conditions. However, when the origin of OTUs was analyzed for specific flow conditions, we detected a strong shift in the soil bacterial community during high flow as compared to low flow. During high flow, 34.4% of bacterial sequences consisted of OTUs that did not occur in soil water during low flow (Fig. 6). These high flow specific soil OTUs were also detected further downstream during high and low flow conditions, respectively. In streams, the fraction of specific high flow soil OTUs accounted for 33% of the sequences originating from soil and 24% of all OTUs detected in streams, independent of flow conditions (Fig. 6). On a phylum level, this fraction appeared to be dominated by Bacteroidetes, Planctomycetes and a combination of ‘Other’ phyla in soils and streams (13%, 16%, and 20% respectively in soils and 11%, 19%, and 18% respectively in streams).

## Discussion

In this study, we explored the effects of hydrological conditions on the soil microbial inoculation to streams, thereby complementing previous studies, which showed the importance of soil microbial inoculation on stream community composition [5, 8, 9] and the impact of flow on the abundance of bacteria that are mobilized from catchment soils [10]. Our results demonstrate that flood events drive bacterial community composition in stream ecosystems. High flow events trigger a different soil microbial inoculation than during low flow conditions that influences aquatic microbial diversity (Fig. 6).

We found that bacterial communities in all habitats were dominated by apparently terrestrially derived OTUs, which on average accounted for > 70% of the sequences identified in aquatic communities and biofilms. This percentage was even higher according to the FEAST analysis. Only a few OTUs were unique to a given habitat type and strong overlaps were identified in the bacterial communities across habitats (Figs. 2 and 6). Additionally, soil indicator OTUs were detected in headwater streams and the OSB water column. This agrees with previous studies, which described a continuity between terrestrial and aquatic communities, suggesting that soils are a part of the stream network metacommunity [8, 9, 14, 21]. In contrast to studies which indicated that the strong physical habitat differences between soils and aquatic habitats could prevent microbes from crossing the terrestrial-aquatic interface [30], our study supports the notion that many soil bacteria are present in the interstitial water between soil particles and could therefore be adapted to a planktonic life in aquatic environments [32]. Furthermore, we observed an increase of bacterial diversity and richness between soil water and streams, which goes in line with the concept of headwater streams being the initial mixing and dispersing zones for communities originating from various upslope terrestrial environments [5, 8, 9].

Community composition in all habitats agreed with earlier findings in freshwater systems typically dominated by Proteobacteria, Bacteroidetes, Actinobacteria, and Cyanobacteria [17, 49]. Alpha-, Beta-, and Gammaproteobacteria appeared to be slightly more abundant in soil water than in downstream habitats in the present study. While this is consistent with earlier studies for Gammaproteobacteria [8], it contrasts with previous findings for Betaproteobacteria, which were found to increase from soil water to downstream habitats [8, 9]. The decreasing abundance of Alphaproteobacteria contradicts findings from a tundra freshwater system (Crump et al. 2012), but agrees with findings from a boreal system (Ruiz-Gonzalez et al. 2015). These diverging findings might indicate a significant influence of local environmental conditions, such as the presence of permafrost, on the bacterial communities. Yet, overall, they agree on the strong observed overlap between soil water and nearby surface waters.

Bacteroidetes showed a decrease from soil water to downstream habitats, thereby contradicting earlier findings [8, 9]. Members of the Bacteroidetes are capable of degrading terrestrial organic matter by breaking down polymeric organic substances, which can be assumed to be advantageous in soil water and headwaters receiving large amounts of terrestrial organic matter [5, 10]. Planctomycetes, which were common in the indicator OTUs for headwater streams, are now recognized to be common in both soil and freshwater habitats [50]. Similar to Bacteroidetes, members of this phylum can efficiently degrade polymeric organic substances [50]. Parcubacteria, which proportions increased during high flow events, were shown to have reduced genomes indicating a specialized, free-living, or parasitic/symbiotic lifestyle [51]. Cyanobacteria were more important in the OSB than in soil water and headwater streams due to higher light availability in this broader stream.

Biofilms held an overall lower diversity than other habitats. Even though the biofilm OTUs were basically a subset of the suspended OSB community and almost all biofilm OTUs were first identified in upslope environments, the community composition was noticeably different from the other habitats and was dominated by Bacteroidetes. This indicates strong shifts in the relative abundance of phyla and is in line with the assumption that the biofilm environment selects for specific OTUs from the stream water [19]. Cyanobacteria are often an important component of stream biofilms exposed to light [52] and have been suggested to shape bacterial diversity and community composition patterns through allelopathy [5]. Members of the Bacteroidetes are known to exhibit gliding motility, which might facilitate the colonization of surfaces and enable them to thrive in biofilms [17].

An earlier study of high flow events in small headwater streams revealed an increase in soil contributions to small streams in terms of bacterial abundances [10]. Here, we show that high flow events trigger an overall increase of richness and diversity in headwater streams and the OSB, and affect the bacterial community composition in all the studied habitats (Figs. 3, 4, and 5). This is likely a mass effect of bacteria coming in large amounts from the soil and homogenizing communities between aquatic habitats [53]. This notion is further supported by the observation of an increased number of soil indicator OTUs in stream water during flood events.

Alternatively, changes in diversity could also be caused by variations in physical and chemical parameters within each habitat [54, 55]. However, water residence times were shown to rarely exceed 2 h in our headwater streams [10], and we argue that this would be too short for relevant bacterial growth and changes in bacterial communities [56]. The increasing proportion of Actinobacteria and Acidobacteria at high flow in streams, especially prominent in the OSB, rather supports a larger soil contribution during high flow events. Indeed, these phyla are known to be more soil-associated [57], even though some studies reported their presence in lakes and streams [e.g., 11]. Accordingly, typical freshwater phyla, such as Verrucomicrobia [58], decreased in proportion during high flow events in streams.

Although bacterial diversity in soils was not found to be significantly different between high flow and low flow conditions (Fig. 3), we noted a high proportion of OTUs that were only detected in soils during high flow events (Fig. 6). We named these OTUs as ‘high flow specific soil taxa’ that accounted for 34% of soil sequences during flood events. Since high flow would likely mobilize particles and increase their abundance in soil water [59], these high flow specific soil OTUs could represent taxa which live preferentially attached to particles. Streambed erosion by water flow might provide a continuous source of particles and attached bacteria stemming from the immediate vicinity of the stream. Particle-attached bacterial communities have repeatedly been found to harbor distinct communities, which differ from the free-living communities in the surrounding water [13, 60, 61].

An alternative explanation for some of our observations regarding enhanced microbial diversity and richness during high flows could also be caused by groundwater fluctuations as a response to rainfall. During high flow events, hydrologic connectivity between surface soil water and shallow groundwater might be established [27, 62], potentially augmenting the soil water community with additional taxa from shallow groundwater. Upwelling and mixing of this groundwater in the streambed could then lead to the transfer of groundwater bacteria to the stream [63]. Indeed, Parcubacteria, which have been observed to be one of the most important bacterial groups in a limestone karst aquifer [64], were important during high flow in the present study potentially indicating groundwater inflow. However, given the steep gradients in the studied systems, such effects are likely less relevant as compared to direct microbial transfer by soil water into streams.

Overall, this study showed that flood events have a fundamental effect on the bacterial diversity and community composition of stream ecosystems. By enhanced soil contributions during such events, bacterial communities across habitats homogenized and diversity increased. These results demonstrate the need to integrate hydrological events into fluvial microbial community studies.

## Data Availability

Sequences are accessible at the National Center for Biotechnology Information Sequence Read Archive under the accession number PRJNA685744.
